# Paediatric Inflammatory Bowel Disease: Clinical Presentation and Disease Location

**DOI:** 10.12669/pjms.334.12926

**Published:** 2017

**Authors:** Danish Abdul Aziz, Maryum Moin, Atif Majeed, Kamran Sadiq, Abdul Gaffar Biloo

**Affiliations:** 1 Dr. Danish Abdul Aziz, MBBS, MRCPCH, FCPS. Senior Instructor, Department of Paediatrics, Aga Khan University Hospital, Karachi, Pakistan; 2 MaryumMoin, Final Year MBBS Medical StudentAga Khan University Hospital, Karachi, Pakistan; 3 Dr. AtifMajeed, MBBS. Instructor, Department of Gastroenterology, Aga Khan University Hospital, Karachi, Pakistan; 4 Dr. Kamran Sadiq, MBBS, FCPS. AssistantProfessor and PaediatricGastroenterologist, Department of Paediatrics, Aga Khan University Hospital, Karachi, Pakistan; 5 Prof. Dr. Abdul GaffarBilloo, MBBS, MRCP, FRCP, Department of Paediatrics, Aga Khan University Hospital, Karachi, Pakistan

**Keywords:** Inflammatory bowel disease (IBD), Ulcerative colitis (UC), Crohn’s Disease (CD), Indeterminate Colitis (IC), Endoscopy, Extra intestinal manifestation

## Abstract

**Objective:**

To determine different clinical presentationsand disease location demarcatedby upper and lower gastrointestinal endoscopyand relevant histopathologyin children diagnosed with inflammatory bowel disease (IBD).

**Methods:**

This is 5 years (2010 to 2015) retrospective studyconducted at the Aga Khan University Hospitalenrolling65admitted children between 6 months to 15years from either gender, diagnosed with IBD on clinical presentation, endoscopy and biopsy. Different clinical presentations at the time of diagnosis were noted in different categories of the disease. All patients underwent upper and lower (up to the terminal ileum) endoscopy with multiple punch biopsies and histologic assessment of mucosal specimens. All endoscopies were done by paediatric gastroenterologists at endoscopy suite of the hospital and all specimens were reported by the pathology department. ESPGHAN revised criteria for the diagnosis of inflammatory bowel disease in children and an adolescent was used to standardize our diagnosis. Extent of disease on endoscopy and relevant histopathology of the biopsy samples were noted at the time of diagnosis. Data was summarized using mean, standard deviation, numbers and percentages for different variables.

**Results:**

Total 56 children were enrolled according to inclusion criteria. There were 34children (61.53%) diagnosed with ulcerative colitis (UC), 10 patients (16.92%) had Crohn’sDisease (CD) and 11 (21.53%) patients were labeled as Indeterminate colitis (IC). Mean age at onset of symptoms was10.03±2.44 and mean age at diagnosis was11.10±2.36. Abdominal pain (80%) and chronic diarrhea (70%) were common symptoms in CD whereas bloody diarrhea (79.41%) and rectal bleeding(64.70%)were common presentation in UC. Patients diagnosed with indeterminate colitis(IC) had similar clinical features as in UC patients. Only 7% patients had some extra-intestinal features in the form of joint pain and/or uveitis. Aspartate aminotransferase level (95.18 ±12.89) was relatively high in patients withCD in comparison with other categories of IBD. Endoscopic findings and relevant histopathology of biopsy samples in UC showed 65% patient had pan-colitis and 13 % with disease restricted to rectum only whereas in CD 70% patient had disease in ileo-colon and only 10 % had involvement of ileum at the time diagnosis.

**Conclusion:**

Patients with UC dominated in our cohort. The most common clinical presentation in UC was bloody diarrhea and rectal bleeding and patients with CDhad abdominal pain and chronic diarrhea as predominant clinical features. Extraintestinal features were uncommon in our cohort. In endoscopic findings, pan-colitis was the mostfrequentfinding in UC and ileo-colonwas common location in CD. IC and UC shared common clinical features and disease location on endoscopy.

## INTRODUCTION

Inflammatory bowel disease (IBD) comprises of twoassociated but distinctive disorders: ulcerative colitis (UC) and Crohn’s disease (CD). IBD is a disease of young people, with a peak incidence in the second and third decades of life. Approximately twenty-five percent of all cases usually present in childhood and adolescence.^1^ UC is a chronic, idiopathic, diffuse mucosal inflammatory disease of the colon whereas CD is a chronic, idiopathic, trans-mural and patchy, inflammation of one or more segments of the gastrointestinal tract; and the term indeterminate colitis (IC) is used for cases of colitis where pathologic findings are not adequate to differentiate between CD and UC.^2^,^3^

Patients with Ulcerative Colitis usually presents with bloody diarrhea, abdominal pain and rectal bleeding. Crohn’s Disease patients predominantly presents with diarrhea and abdominal pain with increasedtendencies to have systemic features likefever, fatigue, reduced appetite and weight loss. Upper and lower gastrointestinal endoscopy along with tissue biopsy for histopathology are crucial in making prompt diagnosis. Incidence of pediatric IBD varies in different countries, the highest being reported in Western countries especially in Northern parts of America and Europe^4^. A growing tendency in the incidence and prevalence of inflammatory bowel disease (IBD) in Asia has been documented for the past two decades. It has been hypothesized that this phenomenon may be associated with the westernization of lifestyles, including changes in dietary practices and environmental changes such as improved hygiene and industrial development.^5^

There is paucity of valid epidemiological studies on IBD from Asian countries and the results are variable. Limited data is available regarding south Asian children with IBD and mostly reported from immigrant population in West.^6^,^7^ This study highlights the different clinical presentations of children with IBD, their endoscopic location and related laboratory data.

## METHODS

A five years retrospective study was conducted at Aga Khan University Hospital from January 2010 to December 2015 after approval from Ethical review committee of the institution. Patients diagnosed with inflammatory bowel disease (IBD) on the basis of clinical history, physical examination, endoscopic and histopathology findings from age 6 months to 15 years from either gender were enrolled in this study. Different clinical presentations (including intestinal and extra-intestinal) at the time of diagnosis were noted in different categories of the disease.

Patients were categorized into three groups depending on the type of pathology seen on biopsy and endoscopy. All patients underwent upper and lower (up to the terminal ileum) endoscopy with multiple punch biopsies and histologic assessment of mucosal specimens. All Endoscopies were done by paediatric gastroenterologist at Endoscopy suite, the Aga Khan University Hospital and all specimens were reported by Pathology department of the hospital. ESPGHAN revised criteria for the diagnosis of inflammatory bowel disease in children and an adolescent was used to standardize our diagnosis.^8^

**Ulcerative Colitis** (UC) was defined by the occurrence of continuous inflammation limited to the colon with histologically typical chronic inflammation limited to the mucosa. The most dependable feature to diagnose UC is continuous mucosal inflammation of the colon, beginning from the rectum, without small bowel involvement, and without granulomas on biopsy. Characteristic macroscopic features of UC include erythema, friability, granularity, purulent exudates, and ulcers that usually lookas superficial small ulcers.

**Crohn’s Disease** (CD) was defined by evidence of a skipped, non-contiguous chronic inflammation of the gastrointestinal tract with or without granulomas and reinforced by clinical, histologic and endoscopic evidence. These key features include the occurrence of skip lesions; the presence of well-formed non-caseating granulomas distant from ruptured crypts any place in the gut; the existence of macroscopic lesions of the upper intestinal tract, in specific serpentine ulcers and cobblestoning, stenosis or structuring of bowel.

**Indeterminate Colitis** (IC) was defined as inflammatory colitis havinguncertain endoscopic and histologic findings making differentiation between UC and CD difficult even after a complete workup. In addition, such patients did not show any evidence of small bowel disease at the time of diagnosis.

Complete blood count, CRP, albumin and serum Alanine aminotransferase level of these enrolled patients were documented. Extent of disease on endoscopy wasnoted at the time of diagnosis. Radiological evidence in the form of barium studies were collected but it was found that most of the patients did not undergo radiological examination. Results were analyzed using SPSS version 20. Data was summarized using mean, standard deviation, numbers and percentages for different variables including sex, age, clinical features and laboratory and endoscopic findings.

## RESULTS

**Fig. 1:**
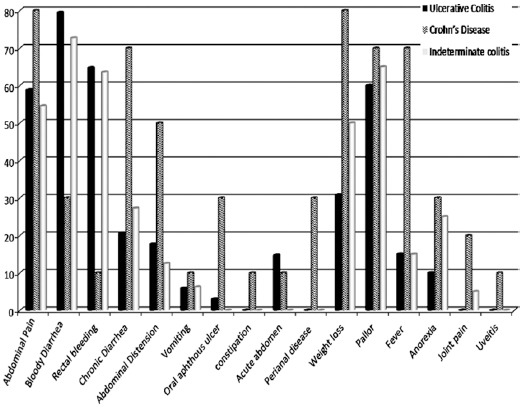
Clinical Presentations.

Total 55 children were enrolled as cases with IBD on the basis of defined criteria. Mean age at onset of disease was 10.03±2.44 and mean age at diagnosis was 11.10±2.36. Thirty nine patients (65%) patient had UC, 10 (16%) patients diagnosed with CD and 16 (21.21%) patients had IC (Table-I). The most common gastrointestinal symptom common in Ulcerative colitis was bloody diarrhea and the most frequent complaints in CD were abdominal pain and chronic diarrhea. No hepatobiliary complications were noted in our study. Only 7% patients had some extra-intestinal features in the form of joint pain and/or uveitis (Fig. 1). Table-II shows disease locations in different categories of IBD.

**Table-I:** Demographic Features.

	*Ulcerative colitis*	*Crohn’s Disease*	*Indeterminate Colitis*	*Total*
	34 (61.81%)	10 (18.18%)	11 (20.00%)	
Male	21(38.18%)	6 (10.90%)	7(12.72%)	34 (61.81%)
Female	13(23.63%)	4(7.27%)	4(7.27%)	21(38.18%)
M: F ratio	1.6 : 1	1.5 : 1	1.7 : 1	1.6 : 1
Median age at onset	10.41 ± 2.61	9.10 ± 1.66	9.72 ± 2.41	10.03±2.44
Median age at diagnosis	11.11 ± 2.47	11.40 ±2.45	10.81 ± 2.08	11.10±2.36
Family History	2 (3.63%)	1(1.81%)	0	3 (5.45%)

**Table-II:** Laboratory Investigations and Mean values with Standard deviation.

*Investigations*	*Ulcerative Colitis*	*Crohn’s Disease*	*Indeterminate Colitis*
Hemoglobin (g/dl)	8.99 ± 1.90	8.53 ± 2.21	9.90 ±2.55
Total leukocyte count (X 10E9/L)	12.35 ±6.30	13.86 ± 5.53	12.40 ± 4.44
Platelet count (X 10E9/L)	220.97 ± 94.48	372.90 ± 166.29	190.09 ± 46.62
C-reactive protein (CRP) mg/dl	2.77 ± 4.62	5.82±6.40	3.23 ± 3.74
Serum Albumin (g/dl)	3.12 ± 1.4	2.98 ± 1.73	3.52 ± 1.81
Alanine aminotransferase (ALT/SGPT) IU/L	34 ± 7.63	95.18 ±12.89	45.90 ± 7.07

**Table-III:** Disease Location.

*Disease and Locations*	*No. of patients*
Ulcerative Colitis (UC)	N=34
Pancolitis	22 (64.70%)
Up to transverse colon	6 (15.38%)
Up to left colon	4(17.94%)
Up to sigmoid Colon	1 (17.94%)
Rectum	1 (12.82%)
Crohn’s Disease (CD)	N= 10
Ileocolon	7 (70.0%)
Colon	1(10.0%)
Terminal ileum	1 (10.0%)
Ileum-colon-upper intestinal tract	1 (5.0%)
Indeterminate Colitis (IC)	N=11
Pancolitis	7 (50.00%)
Up to transverse colon	3 (18.75%)
Up to left colon	3 (18.75%)
Up to sigmoid colon	3(18.75%)
Rectum	0 (0%)

## DISCUSSION

Inflammatory bowel disease is rare pathology in pediatric age group andsomewhat underdiagnosed entity in our region.^9^,^10^ Pediatric IBD is separate entity in its presentation, location of disease and response to treatment. In our cohort male to female ratio was 1.3:1 which is quite similar to the reported studies in pediatric age group. In pediatric CD there is a strong tendency toward males.^11^ Vernier-Massouille et al also confirmed malepredominance, with a similar ratio of 1.4:1 in children younger than 15 years.^12^ Frequencies of different types of IBD vary with age, locationand demography of population. Most studies report a predominance of CD incidence in children and adolescent over UC, although some evidenceshowscontrary picture.^13^ Certain studies proclaimed that the incidence of CD is increasing, particularly in young children, while the UC incidence remains constant.^11^ Van Limburger et al demonstrated a significant predilection for CD in children^14^ whereas some studies showed predominance of UC in pediatric age group.^15^,^16^ Generally UC is found to be more frequent than CD in the preschool age group, whilst CD is three times more frequent than UC in older children in many case series.^17^

Our study is unique in reporting strong predilection towards UC followed by IC. The low frequency of CD is partly due to overlapping symptoms with intestinal tuberculosis which mimics with CD in clinical presentationand disease location on endoscopy.^18^ Intestinal tuberculosis usually presents with an unremitting pattern of ulcers in the colon and small bowel, most characteristically in the ileo-cecal area which is also the most common site for Crohn’s disease.^18^ This also results in late diagnosis in patients with CD as seen in our experience with average delay of two years between onset of symptoms and diagnosis. Clinical presentation correlates with the disease location. Abdominal pain with chronic diarrhea was the most common clinical feature in CD whereas bloody diarrhea, rectal bleeding and abdominal pain were common in UC which are similar to multiple experiences round the globe.^7^,^19^ IC showed similar clinical features to UC in our cohort. Three patients had perianal disease in our series. Pediatric CD mostly behaves with inflammatory or non-stricturing, non-penetrating disease.^20^

In this study only one patient had stricturing disease and onepatient presented with penetrating disease. In our cohort, weight loss followed by fever and pallor were common in patients with CD as compared to UC. Poor growth prior to diagnosis has been documented in many studies examining growth in pediatric IBD.^21^ Extra-intestinal manifestations are reported less frequent in our cohort as in seen in many adults’ IBD series in our region.^19^ In general, extra-intestinal manifestations were more common in Crohn’s disease. Location of the pathology seen on endoscopy is a crucial aspect in diagnosing and characterizing IBD which defines the management and prognosis of the disease. Disease location at presentation varies in pediatric IBD related with adult IBD.^22^ Paediatric UC frequently presents with aggressive phenotype with pancolitis and early time to first surgery compared to adult UC.^20^,^23^

In UC the inflammation may either finish at a transition zone anywhere in the colon or include the entire colon continuously. The most distal part of the terminal ileum may demonstrate non-erosive erythema or edema if there is pancolitis (‘‘backwash ileitis’’).^8^ Inour cohort patients with UC, almost 65% patients had disease involving entire colon. Disease restricted to rectum was seen only in 13% patientswith UC. These findings are quite similar to Sawczenko A et al.^23^ Patients with CD showed most of the patients had presences of disease at ileocolonic area similar to Auvin et al.^20^ Only one patient had terminal ileal disease. In group with IC, 50% patient had involvement of entire colon. In our experience UC and IC had many similarities in the way of clinical presentation and disease location.

## CONCLUSION

Patients with UC dominated in our cohort followed by IC. Frequency of CD was very low in our experience. There is average delay of one year from onset of symptoms and diagnosis including all subtypes of IBD. The most common clinical presentation in UC was bloody diarrhea and rectal bleeding and patients with CD had abdominal pain and chronic diarrhea as predominant clinical features. Extraintestinal features were uncommon in our cohort. In endoscopic findings, pan-cloitis was the most frequent finding in UC and ileo-colon was common location in CD. IC and UC shared common clinical features and disease location on endoscopy.

### Authors Contribution

***DAA:*** Conceptualized and jointly designed the study and drafted the initial manuscript.

***MM and AM:*** Did the data collection, analysis and compiled the results.

***KS and AGB:*** Helped in literature review and in editing of the manuscript for final submission.
